# Aspects of social participation and neighborhood perception: ELSI-Brazil

**DOI:** 10.11606/S1518-8787.2018052000647

**Published:** 2018-10-25

**Authors:** Fabiane Ribeiro Ferreira, Cibele Comini César, Fabíola Bof de Andrade, Paulo Roberto Borges de Souza, Maria Fernanda Lima-Costa, Fernando Augusto Proietti

**Affiliations:** I Universidade Federal de Minas Gerais. Escola de Educação Física, Fisioterapia e Terapia Ocupacional. Departamento de Fisioterapia. Belo Horizonte, MG, Brasil; II Universidade Federal de Minas Gerais. Faculdade de Ciências Econômicas. Centro de Desenvolvimento e Planejamento Regional. Belo Horizonte, MG, Brasil; III Fundação Oswaldo Cruz. Instituto René Rachou. Belo Horizonte, MG, Brasil; IV Fundação Oswaldo Cruz. Instituto de Comunicação e Informação Científica e Tecnológica em Saúde. Rio de Janeiro, RJ, Brasil; V Fundação Oswaldo Cruz. Instituto René Rachou. Programa de Pós-Graduação em Saúde Coletiva. Belo Horizonte, MG, Brasil; VI Fundação Oswaldo Cruz. Instituto René Rachou. Núcleo de Estudos em Saúde Pública e Envelhecimento. Belo Horizonte, MG, Brasil

**Keywords:** Aging, Aged, Social Participation, Socioeconomic Factors, Community Networks, Environment Design, Health Surveys, Envelhecimento, Idoso, Participação Social, Fatores Socioeconômicos, Redes Comunitárias, Planejamento Ambiental, Inquéritos Epidemiológicos

## Abstract

**OBJECTIVE:**

To determine the impact of the physical and social surroundings of the neighborhood, which are presented as facilitators or barriers for the social participation of Brazilian older adults.

**METHODS:**

The study was conducted in a probabilistic representative sample of the Brazilian population aged 50 years and older and who lived in urban areas (n = 7,935). The response variable was social participation, which was defined from two questions about activities performed with other persons: visited friends or relatives in their homes in the last 12 months (yes, no); went out with other persons to public places, such as restaurant, movies, club, park, in the last 12 months (yes, no). The explanatory variables included fear of falling because of defects in sidewalks, concern about the difficulty to get on a bus, subway, or train, difficulty to cross streets, and perception of violence in the neighborhood. Potential confounding variables included age, marital status, education level, self-rated health, living in an asphalted or paved street, time living in the municipality, and socioeconomic position score. Prevalence ratios and respective confidence intervals were estimated using Poisson regression.

**RESULT:**

Difficulty to cross streets presented an independent association with restricted social participation (PR = 0.95; 95%CI 0.93–0.98) among both women (PR = 0.96; 95%CI 0.92–0.99) and men (PR = 0.94; 95%CI 0.90–0.99). Concern about the difficulty to get on a bus, subway, or train was associated with the outcome only among men (PR = 0.95; 95%CI 0.91–0.99). The fear of falling because of defects in sidewalks and the perception of violence in the neighborhood were not associated with social participation.

**CONCLUSIONS:**

Urban characteristics that hinder the crossing of streets and accessibility to public transport can be inferred as important barriers for the social participation of Brazilian older adults.

## RESUMO

**OBJETIVO:**

Determinar o impacto do entorno físico e social da vizinhança, que se apresentam como facilitadores ou barreiras, para a participação social de adultos mais velhos brasileiros.

**MÉTODOS:**

O estudo foi conduzido em amostra probabilística representativa da população brasileira, residente em áreas urbanas, com 50 anos ou mais de idade (n = 7.935). A variável resposta foi participação social, definida a partir de duas perguntas sobre atividades realizadas com outras pessoas: visitou seus amigos ou familiares em suas casas nos últimos 12 meses (sim, não); saiu com outras pessoas para lugares públicos, como restaurante, cinema, clube, praça, nos últimos 12 meses (sim, não). As variáveis explicativas incluíram medo de cair por causa de defeitos nos passeios, preocupação com a dificuldade de subir em ônibus, metrô ou trem, dificuldade em atravessar a rua e percepção de violência na vizinhança. As potenciais variáveis de confusão incluíram faixa etária, situação conjugal, escolaridade, autoavaliação da saúde, residência em rua asfaltada ou pavimentada, tempo de moradia no município e escore de indicador de posição socioeconômica. Razões de prevalência e respectivos intervalos de confiança foram estimados por meio da regressão de Poisson.

**RESULTADO:**

A dificuldade em atravessar a rua apresentou associação independente com a restrição para participação social (RP = 0,95; IC95% 0,93–0,98), tanto entre as mulheres (RP = 0,96; IC95% 0,92–0,99) quanto entre os homens (RP = 0,94; IC95% 0,90–0,99). A preocupação com a dificuldade de subir em ônibus, metrô ou trem apresentou associação com o desfecho somente entre os homens (RP = 0,95; IC95% 0,91–0,99). O medo de cair por causa de defeitos nos passeios e a percepção de violência na vizinhança não foram associados à participação social.

**CONCLUSÕES:**

Características urbanas que ocasionam dificuldade em atravessar a rua e a acessibilidade ao transporte público podem ser inferidas como importantes barreiras para a participação social de adultos mais velhos brasileiros.

## INTRODUCTION

The Brazilian urban aging is a significant social transformation which has aroused great scientific and political interest. Approximately 86% of the older adults currently live in cities^1^, whose physical and social characteristics are manifested as facilitators or barriers to social participation.

Social participation is recognized as a strategy for active aging^2^ ^,^ ^3^, which reinforces the important association between the life scenario and the protagonism of the aging individual. The World Health Organization (WHO) has programs that include social participation, such as Active Ageing^2^, in which it is one of its three pillars, and Age-friendly City^4^, in which it is one of the eight areas to be considered for an accessible city. Furthermore, participation and context are concepts present in the model also proposed by the WHO to explain functionality^5^, which is the paradigm of aging. In this model, participation is presented as one of the components of functionality, which is the result of the interaction of the individual or population with certain health conditions and contextual factors.

Although interest in the construct of social participation is notoriously increasing in several fields of knowledge, there is no consensus as to its definition^6^ ^,^ ^7^, which results in difficulties for peer communication, comparisons between studies, and the definition of assessment instruments^6–8^. Levasseur et al.^8^, after analyzing 43 definitions of social participation, report that it can be defined as the involvement of a person in activities that favor interactions with others in society or community. Six levels of individual involvement are possible in relation to these activities, from the activities of daily living and instrumental activities of daily living to activities that contribute more to the collective, such as participation in political organizations. They propose a distinction between the concepts of participation, social participation, and social engagement based on these six levels of involvement (Figure). Under this perspective, social participation is within a broader concept of participation and adds the construct of social engagement, which would be the set of activities for other persons^6–8^.

**Figure:**
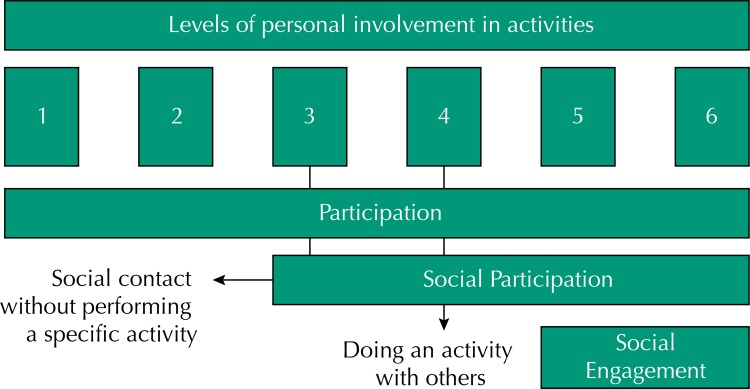
Construction of the response variable according to Levasseur et al.^8^ Brazilian Longitudinal Study of Aging (ELSI-Brazil), 2015–2016.

Social relations in cities, especially those that demand higher levels of involvement, mostly occur in environments built in the physical and social surroundings of neighborhoods in which persons live and move. The characteristics of these spaces and their accessibility are a key factor for the design and favoring of social participation and health^7^ ^,^ ^9^ ^,^ ^10^. Noreau and Bouchen^10^ recognize the context as one of the main factors that influence social participation and that the relations between both are complex and individualized.

In this sense, older individuals are known to be more vulnerable to the characteristics and resources of the urban environment^2–4,9,11^. When these resources and the particularities of individuals interact and negatively impact mobility, the result is the triggering of processes of exclusion^4^. Therefore, the characteristics of the neighborhood can be measured based on the perception of the individuals who enjoy it^9^ ^,^ ^12^ ^,^ ^13^. Attributes typically external to the individual are extremely relevant for the exercise of citizenship, in addition to being potentially modifiable^9^.

Movement in the urban environment is an individual and collective right and an important factor for the social participation of persons with implications for their health^4^ ^,^ ^9^ ^,^ ^10^ ^,^ ^13^. Therefore, the objective of this study is to evaluate the impact of the physical and social surroundings of the neighborhood, understood as facilitators or barriers, for aspects of social participation of Brazilian older adults.

## METHODS

### Study Design

This is a cross-sectional study using the baseline of the Brazilian Longitudinal Study of Aging (ELSI-Brazil). The baseline survey was conducted between 2015 and 2016.

### Sample

The sample was designed to be representative of the community-dwelling Brazilian population aged 50 years and over. The geographical operational base of the Brazilian Institute of Geography and Statistics (IBGE)^14^ was used for the stratification and selection of areas. For the sampling of the ELSI-Brazil, we used different strategies combining the stratification of primary sampling units (municipalities), census tracts, and households. The municipalities were allocated to four strata depending on the size of the population. All residents of the selected households aged 50 years and over were eligible for interview and other procedures. The final sample consisted of 9,412 persons living in 70 municipalities in different Brazilian regions. For this study, the sample was restricted to 7,935 residents of urban areas. More details on the ELSI-Brazil can be found on the research homepage^a^ and in another publication^14^.

The ELSI-Brazil was approved by the Research Ethics Committee of the Oswaldo Cruz Foundation, Minas Gerais (CAAE 34649814.3.0000.5091). Participants signed separate informed consent forms for each following step: interviews, physical measurements, laboratory assays, sample storage, and access to administrative records.

### Variables

#### Social Participation

The construction of the variable of social participation had as a reference the definition suggested by Levasseur et al.^8^, as well as the distinctions between the constructs of participation, social participation, and social engagement, proposed based on the level of involvement of individuals in their interactions. Within this perspective, social participation comprises activities from the third to the sixth level of individual involvement. The interest of this study lies in the aspect of social participation that involves activities practiced with other persons (levels 3 and 4, Figure) instead of activities performed for other persons (social engagement). Therefore, this study measures aspects of social participation and not the construct in its full extent, although the response variable is referred to as social participation.

In this sense, the response variable was the result of the following variables: visited friends or relatives in their homes in the last 12 months (yes, no); went out with other persons to public places, such as restaurant, movies, club, park, in the last 12 months (yes, no). Those who answered *no* for both variables were considered non-participants (reference category) and the others were participants.

Four different variables were independently considered as the main explanatory variables, namely: 1) “When you leave your house, are you afraid of falling because of defects in sidewalks?” (yes, no); 2) “When you leave your house, are you worried about the difficulty to get on a bus, subway, or train?” (yes, no); 3) “When you leave your house, are you worried about the difficulty to cross streets?” (yes, no); 4) “Thinking about crimes and violence, which of the following options better define your neighborhood?” (very safe, safe, very unsafe); we regarded as a safe neighborhood those who reported it as very safe or safe, and as an unsafe neighborhood those who reported it as very unsafe. We read the following definition of neighborhood before to the respondent: “Neighborhood is the place where you live and carry out routine tasks such as going to the bakery, market, fair, local shops, visiting your neighbors, walking. You can also understand neighborhood as the place where you recognize most people.” The explanatory variables are highly correlated (p < 0,0005), data not shown).

We considered as potential confounding variables the following: age group: 50–59, 60–69 and 70–79, 80 years and over; marital status: lives with a partner [marriage, living together, common-law marriage], does not live with a partner [divorced, separated, widow/widowed]; education level: did not go to school, 1–4, 5–8, and 9 years or more; self-rated health (very good or good, fair, poor or very poor); lives in an asphalted or paved street: (yes, no); time living in the municipality in years (< 10; 10–19; 20–29; 30–39; 40–49; 50 or more); and socioeconomic position score, which is constructed from the analysis of the principal component analysis based on the number of household appliances and vehicles and the presence of domestic workers and categorized in quintiles.

#### Statistical analysis

We used the Poisson regression model to determine and quantify the effect of the explanatory variables on social participation. To ensure comparability between the four different regression models, all potential confounding variables were included in the models. The sampling design was incorporated in the analysis using the *svy* command. The magnitudes of the associations were estimated using the prevalence ratio (PR) and respective 95% confidence interval. In all analyses, we used the statistical package Stata version 14.0 (StataCorp LP, College Station, Texas, United States).

## RESULTS

A total of 7,935 residents of urban areas participated in this study, of which 4,516 (56.9%) were female (Table 1). Table 2 presents the results of the univariate analysis of the association between social participation and selected characteristics and respective PR.

**Table 1:** Frequencies related to characteristics selected for older adults (50 years and over). Brazilian Longitudinal Study of Aging (ELSI-Brazil), 2015–2016.

Characteristic	Women	Men	p
		
	%	%	%
Social participation			0.900
Yes	81.8	82.0	
No	18.1	18.0	
Fear of falling because of defects in sidewalks			< 0.001
Yes	60.3	43.4	
No	39.7	56.6	
Difficulty to cross streets			< 0.001
Yes	50.4	35.8	
No	49.6	64.2	
Concern about the difficulty to get on a bus, subway, or train			< 0.001
Yes	42.7	24.1	
No	57.3	75.9	
Perception of violence in the neighborhood			< 0.001
Non-violent	61.5	62.0	
Violent	38.5	38.0	
Age group (years)			0.012
50–59	45.0	51.4	
60–69	30.0	28.9	
70 and over	25.0	19.7	
Marital status			< 0.001
Does not live with a partner	48.2	25.3	
Lives with a partner	51.8	74.7	
Formal education (years)			< 0.001
Did not go to school	12.1	9.2	
1–4	36.3	35.0	
5–8	22.1	24.2	
9 or more	29.5	31.6	
Socioeconomic position score (quintiles)			0.002
1 (worst)	16.4	14.1	
2	20.5	17.2	
3	21.2	20.0	
4	20.6	24.2	
5 (best)	21.3	24.5	
Body mass index			< 0.001
Low weight	6.3	7.5	
Adequate	28.5	33.5	
Overweight	65.2	59.0	
Self-rated health			0.05
Very good/Good	44.8	45.7	
Fair	43.0	44.2	
Poor/Very poor	12.2	10.1	
Lives in an asphalted/paved street			0.05
No	11.5	10.5	
Yes	88.5	89.5	
Time living in the municipality (years)			0.002
< 10	6.3	7.3	
10–19	6.8	7.5	
20–29	10.4	11.0	
30–39	16.1	17.5	
40–49	15.2	17.5	
50 or more	45.2	39.2	

Maximum number of missing information: n = 235 for the variable of perception of violence in the neighborhood in the group of women and n = 148 for the variable of body mass index in the group of men.

**Table 2:** Univariate analysis related to social participation, according to characteristics selected for older adults aged 50 years and over. Brazilian Longitudinal Study of Aging (ELSI-Brazil), 2015–2016.

Characteristic	Social participation		

	Women	Men	Total
		
	PR (95%CI)	PR (95%CI)	PR (95%CI)
Fear of falling because of defects in sidewalks			
No	1.00	1.00	1.00
Yes	1.00 (0.97–1.04)	0.97 (0.94–1.01)	0.99 (0.96–1.02)
Difficulty to cross streets			
No	1.00	1.00	1.00
Yes	0.91(0.88–0.96)*	0.89 (0.85–0.93)*	0.91 (0.88–0.93)*
Concern about the difficulty to get on a bus, subway, or train			
No	1.00	1.00	1.00
Yes	0.93 (0.90–0.97)*	0.88 (0.84–0.92)*	0.92 (0.89–0.95)*
Perception of violence in the neighborhood			
Non-violent	1.00	1.00	1.00
Violent	1.00 (0.96–1.04)	1.03 (0.97–1.07)	1.01 (0.98–1.04)
Age group (years)			
50–59	1.00	1.00	1.00
60–69	0.96 (0.92–1.00)	0.96 (0.91–1.01)	0.96 (0.93–0.99)*
70–79	0.86 (0.80–0.92)*	0.89 (0.83–0.95)*	0.87 (0.83–0.91)*
80 and over	0.85 (0.78–0.94)*	0.76 (0.65–0.88)*	0.82 (0.76–0.88)*
Marital status			
Does not live with a partner	1.00	1.00	1.00
Lives with a partner	1.06 (1.02–1.10)*	1.15 (1.08–1.21)*	1.09 (1.05–1.13)*
Formal education (years)			
Did not go to school	1.00	1.00	1.00
1–4	1.20 (1.13–1.28)*	1.26 (1.13–1.40)*	1.22 (1.15–1.30)*
5–8	1.28 (1.20–1.37)*	1.36 (1.22–1.53)*	1.31 (1.23–1.40)*
9 or more	1.39 (1.29–1.49)*	1.48 (1.33–1.66)*	1.42 (1.33–1.53)*
Socioeconomic position score (quintiles)			
1 (worst)	1.00	1.00	1.00
2	1.15 (1.06–1.25)*	1.10 (1.01–1.18)*	1.13 (1.06–1.20)*
3	1.23 (1.13–1.33)*	1.14 (1.05–1.23)*	1.19 (1.11– 1.26)*
4	1.27 (1.20–1.35)*	1.22 (1.14–1.31)*	1.25 (1.19–1.32)*
5 (best)	1.33 (1.23–1.44)*	1.34 (1.25–1.43)*	1.34 (1.27–1.41)*
Body mass index			
Adequate	1.00	1.00	1.00
Low weight	0.93 (0.83–1.04)	0.96 (0.87–1.05)	0.95 (0.88–1.02)
Obese	0.99 (0.96–1.02)	1.01 (0.96–1.05)	1.00 (0.97–1.03)
Self-rated health			
Very good/Good	1.00	1.00	1.00
Fair	0.94 (0.91–0.98)*	0.93 (0.90–0.97)*	0.94 (0.91–0.97)*
Poor/Very poor	0.81 (0.76–0.88)*	0.78 (0.71–0.85)*	0.80 (0.75–0.85)*
Lives in an asphalted/paved street			
No	1.00	1.00	1.00
Yes	1.02 (0.97–1.09)	1.09 (1.01–1.17)*	1.05 (1.01–1.10)*
Time living in the municipality (years)			
≤ 10	1.00	1.00	1.00
11–20	0.91 (0.85–0.98)*	1.01 (0.91–1.13)	0.96 (0.90–1.03)
> 20	0.93 (0.88–0.99)*	1.02 (0.95–1.10)	0.98 (0.94–1.01)

* p < 0.05

All variables were adjusted for age.

For the study population, we observed statistically significant and positive associations between social participation and persons living with partners, with more years of formal education, with a higher socioeconomic position score, and those living in paved streets. The variables of age and self-rated health also showed a significant but negative association with the response variable. The variables of formal education, socioeconomic position score, and self-rated health showed a gradient of association with social participation, which indicates a dose-response relationship.

The variables of living in paved streets and time living in the municipality were the only ones that differed for men and women, in which the former was significant for the male group and the latter was significant for the female group.

Concerning the variables of interest, the report of difficulty to cross streets and the concern about the difficulty to get on a bus, subway, or train restricted the social participation of all participants regardless of sex, which did not occur for the fear of falling because of defects in sidewalks and the perception of violence in the neighborhood.

The final results of the multivariate analysis can be seen in Table 3. The report of difficulty to cross streets is a limiting factor for the social participation of men and women aged 50 years and over. Concern about the difficulty to use public transport was significant only for men. As in the univariate analysis, the fear of falling because of defects in sidewalks and the perception of violence in the neighborhood were not associated with social participation.

**Table 3:** Final result of the multivariate analysis for social participation related to the explanatory variables selected for older adults aged 50 years and over. Brazilian Longitudinal Study of Aging (ELSI-Brazil), 2015–2016.

Explanatory variable	Social participation		

	Women	Men	Total
		
	PR (95%CI)	PR (95%CI)	PR (95%CI)
Fear of falling because of defects in sidewalks	1.02 (0.99–1.05)	0.99 (0.96–1.03)	1.01 (0.99–1.04)
Difficulty to cross streets	0.95 (0.92–0.99)*	0.94 (0.90–0.99)*	0.96 (0.93–0.98)*
Concern about the difficulty to get on a bus, subway, or train	0.98 (0.95–1.03)	0.95 (0.91–0.99)*	0.98 (0.96–1.00)
Perception of violence in the neighborhood	1.00 (0.96–1.03)	1.03 (0.99–1.08)	1.02 (0.99–1.04)

* p < 0.05

Adjusted for: age group, marital status, formal education, socioeconomic position score, body mass index, self-rated health, living in an asphalted or paved street, and time living in the municipality.

## DISCUSSION

There are several definitions for social participation in the literature^6–8,15^. In this study, social participation was defined from the model of Levasseur et al.^8^ and we chose to delineate the dependent variable according to levels three and four of the individual’s involvement with the activity. Social participation was then measured using two questions that refer to social interaction and living using the meeting of persons without specific activity (visited friends or relatives in their homes in the last 12 months) and carrying out a specific activity (went out with other people to public places, such as restaurant, movies, club, park, in the last 12 months). Thus, we measured an aspect of social participation that does not involve social engagement.

The prevalence of social participation was not different for men and women (81% in both groups), although research shows biological or non-biological contrasts in several aspects of aging between the sexes^16^ ^,^ ^17^. Studies emphasize the importance of recognizing the heterogeneity in the coping strategies used with the challenges to participation including sex differences^18^. In the model of Levasseur et al.^8^, adopted in our study, there are four levels of involvement with the activity, and levels five and six are related to social engagement. According to the authors, social engagement is within social participation. It is possible that the absence of variables that contemplate these levels may have resulted in the similarity of the prevalence of social participation between the sexes and, therefore, different aspects of participation may also present different relations when evaluated separately^15^. However, the objective of this study was to evaluate aspects of social participation that involve activities carried out with others, instead of those carried out for others.

Most of the individuals in this study are female, which brings us back to the current scenario of aging and confirms the feminization of this process, in which women make up most of the population aged 60 years and over and live for up to seven years more than men on average^19^. The IBGE estimates a contingent of 33 million older men and 40.6 million older women for 2060, which means a surplus of 7.6 million women. Explanations for these differences are based on the distinct modes of behavior of men and women throughout life, which are determined by their biological differences and their uneven social trajectories^16^ ^,^ ^17^ ^,^ ^19^, and which reinforces the importance of studies that contemplate non-biological factors.

The univariate analyses showed that, for this group of Brazilian older adults, favorable social participation was associated with factors such as lower age groups, living with a partner, higher formal education, better socioeconomic position, and better self-rated health, in addition to factors related to the place of residence, such as living in paved streets, shorter time living in the municipality (for women only), and not reporting difficulty to cross streets and concern about getting on public transport.

Marital status is an aspect of the life of older adults that is constantly explored in studies on aging^9^ ^,^ ^12^ ^,^ ^20^. On the one hand, living with a partner can increase the social life of older adults and favor their health care and participation. On the other hand, situations such as widowhood and dependency modify the structure of social networks and family relationships over time and can lead to behaviors of isolation in later life^20^, although Donnelly and Hinterlong^21^ have reported the maintenance or even increase of the levels of participation of the older adult after the loss of the spouse as a form of compensation.

Self-rated health has been shown to be a reliable and widely used indicator to estimate the health status, disability, and mortality of older adults^22^. In this study, the prevalence of poor or very poor self-rated health did not vary between the sexes (12%) and was associated with social participation with a dose-response gradient. Silva^23^, in a study with Portuguese older adults, has also found an association between a more positive self-rated health and social participation, volunteering, and cultural activity. At the same time, Sirven and Debrand^24^, with data from the Survey of Health, Ageing and Retirement in Europe (SHARE), have shown that greater social participation can improve health perception among persons aged 50 years and over. The direction of these associations still needs to be better explored^24^. The recognition of the importance of coexistence in society for the health of the individual in the aging process^25^ can be found in the study by Torres et al.^20^, in which variables such as the lack of meetings with friends and dissatisfaction or indifference regarding personal relationships showed a strong association with limitations in the activities of daily living, even after adjustment for social and demographic characteristics, health status, and other indicators of social relations.

In our study, the variables of socioeconomic position and number of years of formal education also presented a dose-response relationship regarding the participation of the older adult, regardless of sex. The socioeconomic position of the population is directly related to the education level; the increase in income and education means greater access to basic services^26^. The questions that operationalized social participation in this study involve activities that promote the meeting of friends and relatives; the second question suggests the use of public places such as restaurant and the movies. Hypothetically, persons with a better socioeconomic position will be able to enjoy more often leisure activities that involve financial investment and possibly present greater social participation within this perspective.

The longer time living in the same municipality could suggest greater social participation of the individual from the opportunity to develop affective bonds. However, this variable was significant only for women and in an inverse manner. Women living in the municipality for less than 10 years reported greater social participation than those living between 10 and 20 years or more than 20 years. Supposedly, since the variable of social participation in this study involves the movement of the individual to the residence of friends and relatives or public places, even when having a satisfactory social network, the individual can be considered non-participatory, which could be explained by other factors related to the individual or their neighborhood. Furthermore, the same temporal factor may indicate that these persons, because of their advancing age, have suffered great losses, which may have contributed to a less active coexistence.

The multivariate evaluation of our study showed that the social participation of men and women is negatively influenced by the report of difficulty to cross streets, which is more frequent among women (58%). A recently published study^27^ on the speed of the gait of older adults in the city of São Paulo has shown that the time of 1.2 m/s of traffic lights that regulate pedestrian crossings does not correspond to the time needed by community-dwelling older adults to safely cross streets, which may contribute to their social isolation, besides promoting accidents^18^ ^,^ ^28^. Virtually 98% of the individuals of the study walk at a slower pace than traffic lights with a positive association for women and persons with low education^27^.

Concern about the difficulty to cross streets was frequently reported by participants in the Age-friendly City survey of the WHO, which was carried out in 33 cities around the world^4^. As previously mentioned, this is also a concern of Brazilian older adults and is associated with the social participation of all participants. The lack of safety to cross streets and intersections is reflected in mortality and morbidity data from external causes, which are released by the Department of Information Technology of the Brazilian Unified Health System (DATASUS)^29^. In the last year (September 2016 to September 2017), 51% of the deaths of pedestrians in traffic in Brazilian cities involved individuals aged 50 years and over. As for hospitalizations for the same cause, they corresponded to 33% for the same age group.

Concern about getting on and off vehicles, although more prevalent among women (42%) than men (25%), was associated with social participation only among men in the multivariate analysis. This result may be associated with gender issues, as it refers to the concern about the need to receive help, which for many men may represent a violation of their masculinity^30^. However, a study in Puerto Rico has found that women were more likely to experience restricted participation because of the lack of accessibility to transportation systems^18^.

Although they were not significantly associated with social participation in this study, it is important to note that concerns about leaving home because of defects in sidewalks (51%) and the perception of a very unsafe neighborhood (37%) are factors identified as barriers to the social participation of older adults in other studies^4^ ^,^ ^9^ ^,^ ^12^. According to the WHO, inadequate sidewalks in urban centers are almost universal problems. The conditions of sidewalks have an impact on the functionality of older adults^9^ ^,^ ^12^ and they affect their movement in cities^4^. In relation to the perception of violence in the neighborhood, it interferes with the motivation of persons to leave their homes, decreases their autonomy, independence, and physical activity, impairs their physical and emotional health, and has a negative impact on social participation^1^ ^,^ ^4^ ^,^ ^9^ ^,^ ^13^.

Some limitations of this study should be mentioned, such as that it is a transversal study, which hinders the establishment of temporality. Another possible limitation is the common source bias, since the participants reported both the outcome and the main explanatory variables. In addition, the dependent variable does not include the parameter of the frequency of the developed activity, which may have overestimated the participation of individuals.

One of the consequences of the social impact of urban aging is the need for the improvement of the urban infrastructure so that persons with functional diversity can continue carrying out their social roles and exercise their citizenship. Both questions asked to measure social participation in this study refer to the movement need of the individual using the urban space. The planning and organization of cities and their spaces must consider accessibility for the exercise of the collective right of participation, regardless of personal characteristics such as age^2^ ^,^ ^4^ ^,^ ^9^.

While the mobility of older adults refers to their ability to move and carry out tasks outside home, urban mobility is related to attributes of the urban environment that favor the movement of persons to carry out various activities in the city using vehicles, mobility aids, and walking. Crossing streets and intersections, getting on and off vehicles, and safely walking on sidewalks are essential tasks for mobility and consequent social participation^4^ ^,^ ^18^ ^,^ ^27^ ^,^ ^28^.

Given the above, it is irrefutable that social participation is a construct inseparable from the context of cities, even if their relations are complex and still need to be explored. The data analyzed in this study are representative of the Brazilian population. Therefore, urban characteristics that hinder the crossing of streets and accessibility to public transport can be inferred as important barriers for the social participation of Brazilian older adults.

## Footnotes

a Fundação Oswaldo Cruz. Brazilian Longitudinal Study of Aging. Rio de Janeiro; c2015 [cited 2017 Nov 28]. Available from: http://elsi.cpqrr.fiocruz.br
